# Supplementary material for: Identification and Characterization of Neofusicoccum stellenboschiana in Branch and Twig Dieback-Affected Olive Trees in Italy and Comparative Pathogenicity with N. mediterraneum
Source: J Fungi (Basel). 2023 Feb 23;9(3):292. doi: 10.3390/jof9030292 (PMC10053632; doi:10.3390/jof9030292)
Supplement: Supplementary file 1 [file jof-09-00292-s001.zip › Figure_S1.pdf]

Title:
Identification and characterization of *Neofusicoccum stellenboschiana* in branch and twig dieback-affected olive trees in Italy and comparative pathogenicity with *N. mediterraneum*

Journal name:
Journal of Fungi - MDPI

Authors:
Giuliano Manetti, Angela Brunetti, Valentina Lumia, Lorenzo Sciarroni, Paolo Marangi, Nicola Cristella, Francesco Faggioli, Massimo Reverberi, Marco Scortichini and Massimo Pilotti

Corresponding author:
Massimo Pilotti

Affiliation:
Institution: Council for Agricultural Research and Economics; Department: Research Center for Plant Protection and Certification (CREA-DC) - Via C. G. Bertero 22, 00156 Rome, Italy

E-mail address: massimo.pilotti@crea.gov.it

Figure S1 Phylogeny of the *Neofusicoccum* genus, based on ITS + TEF1-α + TUB2 + RPB2 data set and including the botryosphaeriaceous isolates from Apulia, Latium and Tuscany (Italy), CREA-DC TPR OL.431, 438, 60, 453 (shaded in blue-sky within a red frame). The phylogeny was inferred using the ML method and General Time Reversible model [34], with 1000 bootstraps. The evolutionary history was inferred by using the Maximum Likelihood method and General Time Reversible model [34]. The tree with the highest log likelihood (-10197.21) is shown. The percentage of trees in which the associated taxa clustered together is shown next to the branches (values > 40). Initial tree(s) for the heuristic search were obtained automatically by applying Neighbor-Join and BioNJ algorithms to a matrix of pairwise distances estimated using the Maximum Composite Likelihood (MCL) approach, and then selecting the topology with superior log likelihood value. This analysis involved 78 nucleotide sequences. There were a total of 2420 positions in the final dataset. Evolutionary analyses were conducted in MEGA X [33]

References

33. Kumar, S.; Stecher, G.; Li, M.; Knyaz, C.; Tamura, K. MEGA X: molecular evolutionary genetics analysis across computing platforms. Mol. Biol. Evol. 2018, 35(6), 1547-1549. Doi: 10.1093/molbev/msy096.
34. Nei, M.; Kumar, S. Molecular evolution and phylogenetics. Oxford University Press Inc., Oxford, 2000; pp. 333.

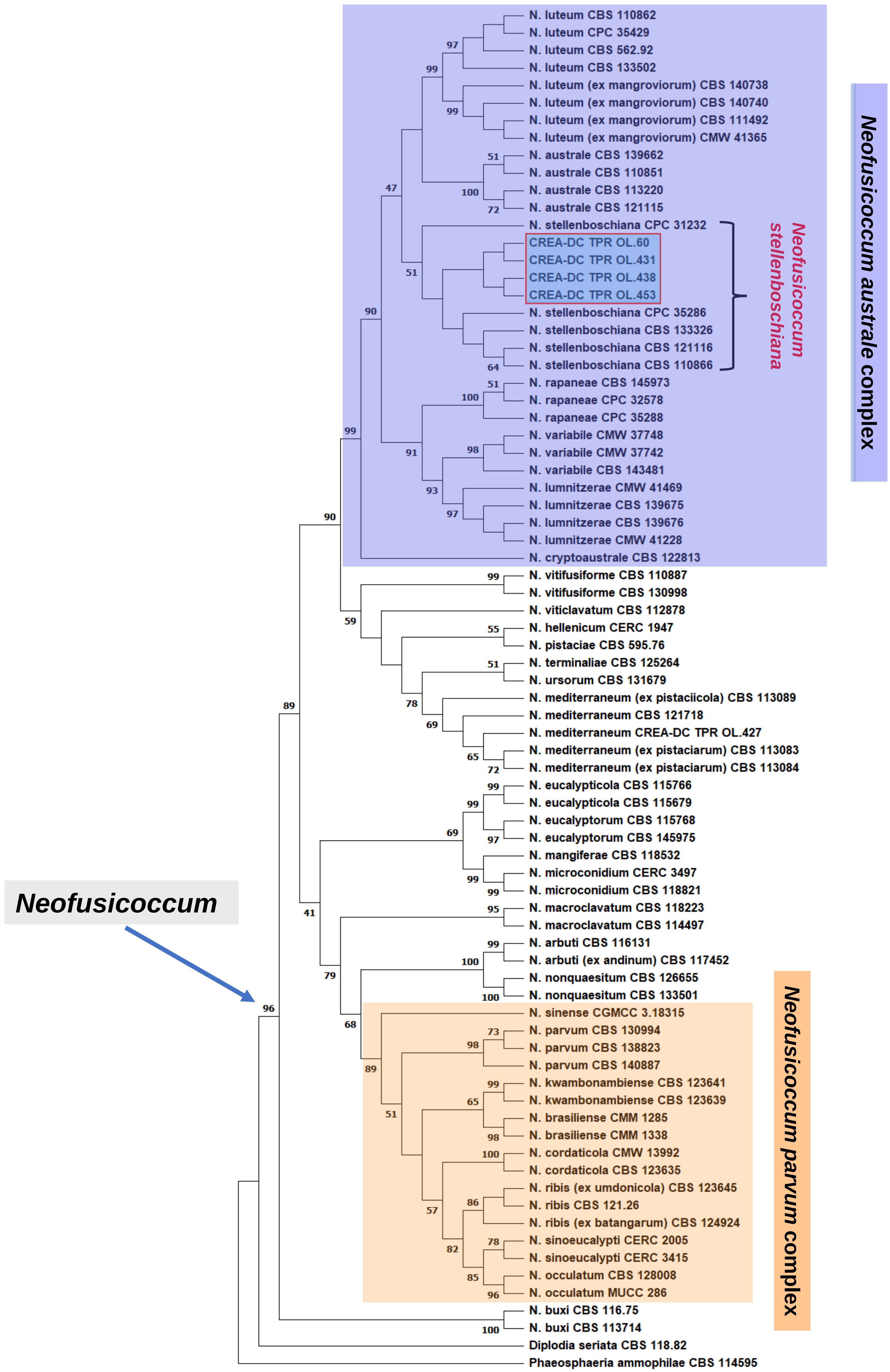

N. luteum CBS 110862
N. luteum CPC 35429
N. luteum CBS 562.92
N. luteum CBS 133502
N. luteum (ex mangroviorum) CBS 140738
N. luteum (ex mangroviorum) CBS 140740
N. luteum (ex mangroviorum) CBS 111492
N. luteum (ex mangroviorum) CMW 41365
N. australe CBS 139662
N. australe CBS 110851
N. australe CBS 113220
N. australe CBS 121115
N. stellenboschiana CPC 31232
CREA-DC TPR OL.60
CREA-DC TPR OL.431
CREA-DC TPR OL.438
CREA-DC TPR OL.453
N. stellenboschiana CPC 35286
N. stellenboschiana CBS 133326
N. stellenboschiana CBS 121116
N. stellenboschiana CBS 110866
Neofusicoccum stellenboschiana
N. rapaneae CBS 145973
N. rapaneae CPC 32578
N. rapaneae CPC 35288
N. variabile CMW 37748
N. variabile CMW 37742
N. variabile CBS 143481
N. lumnitzerae CMW 41469
N. lumnitzerae CBS 139675
N. lumnitzerae CBS 139676
N. lumnitzerae CMW 41228
N. cryptoaustrale CBS 122813
Neofusicoccum australe complex
N. vitifusiforme CBS 110887
N. vitifusiforme CBS 130998
N. viticlavatum CBS 112878
N. hellenicum CERC 1947
N. pistaciae CBS 595.76
N. terminaliae CBS 125264
N. ursorum CBS 131679
N. mediterraneum (ex pistaciicola) CBS 113089
N. mediterraneum CBS 121718
N. mediterraneum CREA-DC TPR OL.427
N. mediterraneum (ex pistaciarum) CBS 113083
N. mediterraneum (ex pistaciarum) CBS 113084
N. eucalypticola CBS 115766
N. eucalypticola CBS 115679
N. eucalyptorum CBS 115768
N. eucalyptorum CBS 145975
N. mangiferae CBS 118532
N. microconidium CERC 3497
N. microconidium CBS 118821
N. macroclavatum CBS 118223
N. macroclavatum CBS 114497
N. arbuti CBS 116131
N. arbuti (ex andinum) CBS 117452
N. nonquaesitum CBS 126655
N. nonquaesitum CBS 133501
Neofusicoccum
N. sinense CGMCC 3.18315
N. parvum CBS 130994
N. parvum CBS 138823
N. parvum CBS 140887
N. kwambonambiense CBS 123641
N. kwambonambiense CBS 123639
N. brasiliense CMM 1285
N. brasiliense CMM 1338
N. cordaticola CMW 13992
N. cordaticola CBS 123635
N. ribis (ex umdonicola) CBS 123645
N. ribis CBS 121.26
N. ribis (ex batangarum) CBS 124924
N. sinoeucalypti CERC 2005
N. sinoeucalypti CERC 3415
N. occulatum CBS 128008
N. occulatum MUCC 286
Neofusicoccum parvum complex
N. buxi CBS 116.75
N. buxi CBS 113714
Diplodia seriata CBS 118.82
Phaeosphaeria ammophilae CBS 114595
