# Supplementary material for: Identification and Characterization of Neofusicoccum stellenboschiana in Branch and Twig Dieback-Affected Olive Trees in Italy and Comparative Pathogenicity with N. mediterraneum
Source: J Fungi (Basel). 2023 Feb 23;9(3):292. doi: 10.3390/jof9030292 (PMC10053632; doi:10.3390/jof9030292)
Supplement: Supplementary file 1 [file jof-09-00292-s001.zip › Table_S1.pdf]

Title:

Identification and characterization of *Neofusicoccum stellenboschiana* in branch and twig dieback-affected olive trees in Italy and comparative pathogenicity with *N. mediterraneum*

Journal name:

Journal of Fungi - MDPI

Authors:

Giuliano Manetti, Angela Brunetti, Valentina Lumia, Lorenzo Sciarroni, Paolo Marangi, Nicola Cristella, Francesco Faggioli, Massimo Reverberi, Marco Scortichini and Massimo Pilotti

Corresponding author:

Massimo Pilotti

Affiliation:

Institution: Council for Agricultural Research and Economics; Department: Research Center for Plant Protection and Certification (CREA-DC) - Via C. G. Bertero 22, 00156 Rome, Italy

E-mail address:

massimo.pilotti@crea.gov.it

Table S1. Percentage of nucleotide identity of sequences of Italian isolates of *Neofusicoccum stellenboschiana* with sequences of reference CBS strains of the same species.

Reference sequences (DNA locus)	*Neofusicoccum stellenboschiana* Italian isolates			
***Neofusicoccum stellenboschiana* type strains (ITS)**	**OL. 453**	**OL.60**	**OL.431**	**OL.438**
CBS_110864, AY343407.1 *	100	100	100	100
CBS_121116, EF445356.1	99.80	99.80	99.80	99.80
CBS_133326, MT587519.1	99.80	99.80	99.80	99.80
CBS_110866, MT587518.1	99.80	99.80	99.80	99.80
**Neofusicoccum stellenboschiana type strains (TEF1-alpha)**	**OL. 453**	**OL.60**	**OL.431**	**OL.438**
CBS_110864 AY343348.1 *	99.60	99.60	99.60	99.60
CBS_121116 EF445387.1	100	100	100	100
CBS_133326 MT592244.1	100	100	100	100
CBS_110866 MT592242.1	100	100	100	100
**Neofusicoccum stellenboschiana type strains (TUB2)**	**OL. 453**	**OL.60**	**OL.431**	**OL.438**
CBS_110864 KX465047.1 *	100	100	100	100
CBS_121116 KX465049.1	100	100	100	100
CBS_133326 MT592736.1	100	100	100	100
CBS_110866 MT592734.1	100	100	100	100
**Neofusicoccum stellenboschiana type strains (RPB2)**	**OL. 453**	**OL.60**	**OL.431**	**OL.438**
CBS_110864 KX464042.1 *	100	100	100	99.83
CBS_121116 KX464044.1	100	100	100	99.83
CBS_133326 MT592433.1	100	100	100	99.83
CBS_110866 MT592431.1	100	100	100	99.83

* The first CBS strain used to establish *Neofusicoccum stellenboschiana* species [31] and previously classified as *N. australe.*

References

31. Yang T.; Johannes Z.; Groenewald J. Z.; Cheewangkoon R,; Jami F.; Abdollahzadeh J.; Lombard L.; Crous P. W. Families, genera, and species of Botryosphaeriales. *Fungal Biol*. **2017**, *121*, 322-346. Doi: 10.1016/j.funbio.2016.11.001.
