# Supplementary material for: Identification and Characterization of Neofusicoccum stellenboschiana in Branch and Twig Dieback-Affected Olive Trees in Italy and Comparative Pathogenicity with N. mediterraneum
Source: J Fungi (Basel). 2023 Feb 23;9(3):292. doi: 10.3390/jof9030292 (PMC10053632; doi:10.3390/jof9030292)
Supplement: Supplementary file 1 [file jof-09-00292-s001.zip › Text_Table_S1.pdf]

Title:

Identification and characterization of *Neofusicoccum stellenboschiana* in branch and twig dieback-affected olive trees in Italy and comparative pathogenicity with *N. mediterraneum*

Journal name:

Journal of Fungi - MDPI

Authors:

Giuliano Manetti, Angela Brunetti, Valentina Lumia, Lorenzo Sciarroni, Paolo Marangi, Nicola Cristella, Francesco Faggioli, Massimo Reverberi, Marco Scortichini and Massimo Pilotti

Corresponding author:

Massimo Pilotti

Affiliation:

Institution: Council for Agricultural Research and Economics; Department: Research Center for Plant Protection and Certification (CREA-DC) - Via C. G. Bertero 22, 00156 Rome, Italy

E-mail address:

massimo.pilotti@crea.gov.it

Text Table S1 Methods for DNA extraction, PCR amplifications and sequencing of DNA regions of *Neofusicoccum stellenboschiana* isolated from olive trees in Apulia, Latium and Tuscany (Italy). In Table A the DNA regions amplified and primers used are reported. In Table B the thermal cycling for each DNA region is described. Below sequences generated in this study and their accession numbers are also listed.

Genomic DNA (gDNA) of fungal isolates was obtained from 100 mg mycelium sampled from axenic cultures actively growing on sterile cellophane discs (BIO-RAD, Hercules, CA, USA) layered on PDA plates and collected by scraping with a sterile pipette tip. Mycelium samples were powdered with liquid nitrogen, then gDNA was extracted using a DNeasy Plant Mini Kit (Qiagen, Hilden, Germany) following the manufacturer's instructions.

PCR amplifications were carried out on a PCR T100™ Thermal Cycler (BIO-RAD). Reaction assembly was in 50µl volume and contained each primer at 0.2 µM, 2.5 U of High Fidelity Platinum Taq DNA Polymerase (Invitrogen, Thermo Fisher Scientific, Waltham, MA, USA), 0.2 mM (ITS) or 0.4 mM (TUB2 e TEF1-α) of each dNTP, in the buffer supplied by the Taq polymerase manufacturer.

Primer sequences and citations are reported in table A. Thermal cycling is reported in table B.

The PCR products were analysed by agarose gel electrophoresis, excised from gel, and eluted with ISOLATE II PCR and gel kit (Meridian Bioscience, River Hills Drive, Cincinnati, OH, USA) following the manufacturer's instructions. Then amplicons were directly sequenced in both directions by Sanger technology (Bio-Fab research s.r.l. Rome Italy).

Table A. DNA regions amplified and primers used

DNA region	Forward primer	Sequence	Reverse primer	Sequence	Citation
ITS	ITS5	5′-GGAAGTAAAAGTCGTAACAAGG-3′	ITS4	5′-TCCTCCGCTTATTGATATGC-3′	[25]
TEF1-α	EF688F	5′-CGGTCACTTGATCTACAAGTG C-3′	EF1251R	5′-CCTCGAACTCACCAGTACCG-3′	[26]
TUB2	T1-β tubulin[1]	5′-AACATGCGTGAGATTGTAAGT-3′	Bt2b[2]	5′-ACCCTCAGTGTAGTGACCCTTGGC-3′	[1] [27] [2] [28]
RPB2	fRPB2-6F	5′-TGGGGKWTGGTYTGYCCTGC-3′	fRPB2-7cR	5′-CCCATRGCTTGYTTRCCCAT-3′	[29]

Table B. Thermal cycling (35 cycles) to amplify DNA regions used for fungal identification (Blast and phylogeny)

Loci DNA	Initial denaturation	Denaturation for each cycle	Annealing	Extension	Final extension
ITS	94°C, 3min	94°C, 30s	50°C, 30s	68°C, 1min 15s	68°C, 10min
TEF1-α	94°C, 3min	94°C, 30s	55°C, 45s	68°C, 1min 30s	68°C, 10min
TUB2	94°C, 3min	94°C, 30s	50°C, 30s	68°C, 1min	68°C, 10min
RPB2	94°C, 3min	94°C, 40s	55°C, 30s	68°C, 1min	68°C, 10min

References

25. White, T.J.; Bruns, T.; Lee, S.; Taylor, J. Amplification and direct sequencing of fungal ribosomal RNA genes for phylogenetics. In *PCR protocols: a guide to methods and applications*. Innis, M.A., Gelfand, D.H., Sninsky, J.J., White, T.J., Eds.; Academic Press Inc.: London, United Kingdom, 1990; pp. 315-322. Doi: 10.1016/B978-0-12-372180-8.50042-1.
26. Alves, A.; Crous, P.W.; Correia, A.; Phillips, A.J.L. Morphological and molecular data reveal cryptic speciation in Lasiodiplodia theobromae. *Fungal Divers.* **2008,** *28,* 1-13.
27. Glass, N.L.; Donaldson, G. Development of primer sets designed for use with PCR to amplify conserved genes from filamentous ascomycetes. *Appl. Environ. Microbiol.* **1995,** *61,* 1323-1330. Doi: 10.1128/aem.61.4.1323-1330.1995.
28. O'Donnell, K.; Cigelnik, E. Two divergent intragenomic rDNA ITS2 types within a monophyletic lineage of the fungus fusarium are nonorthologous. *Mol. Phylogenet. Evol.* **1997,** *7(1),* 103-116. Doi: 10.1006/mpev.1996.0376.
29. Liu, Y.J.; Whelen S.; Hall B.D. Phylogenetic relationships among ascomycetes: evidence from an RNA polymerse II subunit. *Molecular Biol. Evol.* **1999,** *16,* 1799–1808. Doi: 10.1093/oxfordjournals.molbev.a026092.

Sequences

>accession Nos_fungal isolate code DNA locus:

ITS

>OP893662_CREA-DC TPR OL.60 ITS
TTTCCGTAGGTGAACCTGCGGAAGGATCATTACCGAGTTGATTCGAGCTCCGGCTCGACTCTCCCACCCCATG
TGTACCTACCTCTGTTGCTTTGGCGGGCCGCGGTCCTCCGCACCGACCCCCGTTCGGGGGCCGGCCAGCGCCC
GCCAGAGGACCACAAAACTCCAGTCAGTAAACGTCGCAGTCTGAGAAACAAGTTAATAAACTAAAACTTTCAA
CAACGGATCTCTTGGTTCTGGCATCGATGAAGAACGCAGCGAAATGCGATAAGTAATGTGAATTGCAGAATTC
AGTGAATCATCGAATCTTTGAACGCACATTGCGCCCCTTGGTATTCCGAGGGGCATGCCTGTTCGAGCGTCAT
TTCAACCCTCAAGCTCTGCTTGGTATTGGGCTCCGTCCTCCGCGGACGCGCCTCGAAGACCTCGGCGGTGGCG
TCTTGCCTCAAGCGTAGTAGAAAACACCTCGCTTTGGAGCGCACGGCGTCGCCCGCCGGACGAACCTTTGAAT
TTTTCTCAAGGTTGACCTCGGATCAGGTAGGGATACCCGCTGAACTTAA

>OP893663_CREA-DC TPR OL.431 ITS
TTTCCGTAGGTGAACCTGCGGAAGGATCATTACCGAGTTGATTCGAGCTCCGGCTCGACTCTCCCACCCCATG
TGTACCTACCTCTGTTGCTTTGGCGGGCCGCGGTCCTCCGCACCGACCCCCGTTCGGGGGCCGGCCAGCGCCC
GCCAGAGGACCACAAAACTCCAGTCAGTAAACGTCGCAGTCTGAGAAACAAGTTAATAAACTAAAACTTTCAA
CAACGGATCTCTTGGTTCTGGCATCGATGAAGAACGCAGCGAAATGCGATAAGTAATGTGAATTGCAGAATTC
AGTGAATCATCGAATCTTTGAACGCACATTGCGCCCCTTGGTATTCCGAGGGGCATGCCTGTTCGAGCGTCAT
TTCAACCCTCAAGCTCTGCTTGGTATTGGGCTCCGTCCTCCGCGGACGCGCCTCGAAGACCTCGGCGGTGGCG
TCTTGCCTCAAGCGTAGTAGAAAACACCTCGCTTTGGAGCGCACGGCGTCGCCCGCCGGACGAACCTTTGAAT
TTTTCTCAAGGTTGACCTCGGATCAGGTAGGGATACCCGCTGAACTTAA

>OP893664_CREA-DC TPR OL.438 ITS
TTTCCGTAGGTGAACCTGCGGAAGGATCATTACCGAGTTGATTCGAGCTCCGGCTCGACTCTCCCACCCCATG
TGTACCTACCTCTGTTGCTTTGGCGGGCCGCGGTCCTCCGCACCGACCCCCGTTCGGGGGCCGGCCAGCGCCC
GCCAGAGGACCACAAAACTCCAGTCAGTAAACGTCGCAGTCTGAGAAACAAGTTAATAAACTAAAACTTTCAA
CAACGGATCTCTTGGTTCTGGCATCGATGAAGAACGCAGCGAAATGCGATAAGTAATGTGAATTGCAGAATTC
AGTGAATCATCGAATCTTTGAACGCACATTGCGCCCCTTGGTATTCCGAGGGGCATGCCTGTTCGAGCGTCAT
TTCAACCCTCAAGCTCTGCTTGGTATTGGGCTCCGTCCTCCGCGGACGCGCCTCGAAGACCTCGGCGGTGGCG
TCTTGCCTCAAGCGTAGTAGAAAACACCTCGCTTTGGAGCGCACGGCGTCGCCCGCCGGACGAACCTTTGAAT
TTTTCTCAAGGTTGACCTCGGATCAGGTAGGGATACCCGCTGAACTT
AA

>OP893665_CREA-DC TPR OL.453 ITS
TTTCCGTAGGTGAACCTGCGGAAGGATCATTACCGAGTTGATTCGAGCTCCGGCTCGACTCTCCCACCCCATG
TGTACCTACCTCTGTTGCTTTGGCGGGCCGCGGTCCTCCGCACCGACCCCCGTTCGGGGGCCGGCCAGCGCCC
GCCAGAGGACCACAAAACTCCAGTCAGTAAACGTCGCAGTCTGAGAAACAAGTTAATAAACTAAAACTTTCAA
CAACGGATCTCTTGGTTCTGGCATCGATGAAGAACGCAGCGAAATGCGATAAGTAATGTGAATTGCAGAATTC
AGTGAATCATCGAATCTTTGAACGCACATTGCGCCCCTTGGTATTCCGAGGGGCATGCCTGTTCGAGCGTCAT
TTCAACCCTCAAGCTCTGCTTGGTATTGGGCTCCGTCCTCCGCGGACGCGCCTCGAAGACCTCGGCGGTGGCG
TCTTGCCTCAAGCGTAGTAGAAAACACCTCGCTTTGGAGCGCACGGCGTCGCCCGCCGGACGAACCTTTGAAT
TTTTCTCAAGGTTGACCTCGGATCAGGTAGGGATACCCGCTGAACTTAA

translation elongation factor 1-alpha (TEF1-alpha)

>OQ091952_CREA-DC TPR OL.60 TEF1-alpha
GGTGGTATCGACAAGCGTACCATCGAGAAGTTCGAGAAGGTGAGAAGATTTTTCGTTCCGCTGCCCGCGCGAT
GGTGCTGGGATGCCGCACTCAATTTGTCTTATCGCTTCGGTGAGGGGCATTTTGGTGGTGGGGTCGGCCCGCG
CTAAGCCTCGTTTGTGCTTCGGCAAAATCTCCGCATCTGGTTTTTTGCGACCGGCGTGCGACCGAAGCGCGCC
CCTCGCCAGACTCGCAGCCACACATGTGCGATCGGACGCTAACGACCGTCTCAGGAAGCTGCCGAGCTCGGCA
AGGGTTCCTTCAAGTACGCCTGGGTCCTTGACAAGCTCAAGGCTGAGCGTGAGCGTGGTATCACCATCGACAT
TGCCCTCTGGAAGTTCGAGACCCCGAAGTACTATGTCACCGTCATCGACGCCCCCGGTCACCGTGATTTCATC
AAGAACATGATCACTGGTACCTCCCAGGCCGACTGCGCCATTCTCATCATTGCCGC

>OQ091953_CREA-DC TPR OL.431 TEF1-alpha
GGTGGTATCGACAAGCGTACCATCGAGAAGTTCGAGAAGGTGAGAAGATTTTTCGTTCCGCTGCCCGCGCGAT
GGTGCTGGGATGCCGCACTCAATTTGTCTTATCGCTTCGGTGAGGGGCATTTTGGTGGTGGGGTCGGCCCGCG
CTAAGCCTCGTTTGTGCTTCGGCAAAATCTCCGCATCTGGTTTTTTGCGACCGGCGTGCGACCGAAGCGCGCC
CCTCGCCAGACTCGCAGCCACACATGTGCGATCGGACGCTAACGACCGTCTCAGGAAGCTGCCGAGCTCGGCA
AGGGTTCCTTCAAGTACGCCTGGGTCCTTGACAAGCTCAAGGCTGAGCGTGAGCGTGGTATCACCATCGACAT
TGCCCTCTGGAAGTTCGAGACCCCGAAGTACTATGTCACCGTCATCGACGCCCCCGGTCACCGTGATTTCATC
AAGAACATGATCACTGGTACCTCCCAGGCCGACTGCGCCATTCTCATCATTGCCGC

>OQ091954_CREA-DC TPR OL.438 TEF1-alpha
GGTGGTATCGACAAGCGTACCATCGAGAAGTTCGAGAAGGTGAGAAGATTTTTCGTTCCGCTGCCCGCGCGAT
GGTGCTGGGATGCCGCACTCAATTTGTCTTATCGCTTCGGTGAGGGGCATTTTGGTGGTGGGGTCGGCCCGCG
CTAAGCCTCGTTTGTGCTTCGGCAAAATCTCCGCATCTGGTTTTTTGCGACCGGCGTGCGACCGAAGCGCGCC
CCTCGCCAGACTCGCAGCCACACATGTGCGATCGGACGCTAACGACCGTCTCAGGAAGCTGCCGAGCTCGGCA
AGGGTTCCTTCAAGTACGCCTGGGTCCTTGACAAGCTCAAGGCTGAGCGTGAGCGTGGTATCACCATCGACAT
TGCCCTCTGGAAGTTCGAGACCCCGAAGTACTATGTCACCGTCATCGACGCCCCCGGTCACCGTGATTTCATC
AAGAACATGATCACTGGTACCTCCCAGGCCGACTGCGCCATTCTCATCATTGCCGC

>OQ091955_CREA-DC TPR OL.453 TEF1-alpha
GGTGGTATCGACAAGCGTACCATCGAGAAGTTCGAGAAGGTGAGAAGATTTTTCGTTCCGCTGCCCGCGCGAT
GGTGCTGGGATGCCGCACTCAATTTGTCTTATCGCTTCGGTGAGGGGCATTTTGGTGGTGGGGTCGGCCCGCG
CTAAGCCTCGTTTGTGCTTCGGCAAAATCTCCGCATCTGGTTTTTTGCGACCGGCGTGCGACCGAAGCGCGCC
CCTCGCCAGACTCGCAGCCACACATGTGCGATCGGACGCTAACGACCGTCTCAGGAAGCTGCCGAGCTCGGCA
AGGGTTCCTTCAAGTACGCCTGGGTCCTTGACAAGCTCAAGGCTGAGCGTGAGCGTGGTATCACCATCGACAT
TGCCCTCTGGAAGTTCGAGACCCCGAAGTACTATGTCACCGTCATCGACGCCCCCGGTCACCGTGATTTCATC
AAGAACATGATCACTGGTACCTCCCAGGCCGACTGCGCCATTCTCATCATTGCCGC

beta-tubulin 2(TUB2)

>OQ091956_CREA-DC TPR OL.60 TUB2
ACCTGCGCCTGCCCGCTTGTTTTTCTACGCGTCCCCTGACAGCCCCGCGTCTTGCCCCCCGGCTACGACAACG
GGGTCAACACCGCTGCGCACTCCTGCTAACGTCGTCTTTTCGCATCCATAGGTTCACCTCCAGACCGGCCAAT
GCGTAAGTCTCCTCACATCCGCTGGAATCGCTGCATCGCGCTGACTTTGCCCAGGGTAACCAAATCGGTGCTG
CCTTCTGGTTTGTTGCCAAAACACTGCCGCTCCCGCGCCCCCGCTGACGCGAATCGACACCGCAGGCAGACCA
TTTCTGGCGAGCACGGCCTGGACGGCTCTGGCGTGTAAGTCTGCGCCGTTTCTTGCGCGAATGGCAATGGCTG
ACCCGCAGCAGCTACAATGGCACCTCCGACCTCCAGCTCGAGCGCATGAACGTCTACTTCAACGAGGTACTCT
CTCACTAATCGCACAAACACGTAAAGTATGGCAATCTTCTGAACGCGCAGCAGGCGTCGAACAACAAGTACGT
TCCTCGTGCCGTCCTCGTCGACCTCGAGCCCGGCACCATGGATGCCGTCCGCGCCGGCCCCTTCGGCCAGCTC
TTCCGCCCCGACAACTTTGTCTTCGGCCAGTCTGGTGCCGGTAACAACTGGG

>OQ091957_CREA-DC TPR OL.431 TUB2
ACCTGCGCCTGCCCGCTTGTTTTTCTACGCGTCCCCTGACAGCCCCGCGTCTTGCCCCCCGGCTACGACAACG
GGGTCAACACCGCTGCGCACTCCTGCTAACGTCGTCTTTTCGCATCCATAGGTTCACCTCCAGACCGGCCAAT
GCGTAAGTCTCCTCACATCCGCTGGAATCGCTGCATCGCGCTGACTTTGCCCAGGGTAACCAAATCGGTGCTG
CCTTCTGGTTTGTTGCCAAAACACTGCCGCTCCCGCGCCCCCGCTGACGCGAATCGACACCGCAGGCAGACCA
TTTCTGGCGAGCACGGCCTGGACGGCTCTGGCGTGTAAGTCTGCGCCGTTTCTTGCGCGAATGGCAATGGCTG
ACCCGCAGCAGCTACAATGGCACCTCCGACCTCCAGCTCGAGCGCATGAACGTCTACTTCAACGAGGTACTCT
CTCACTAATCGCACAAACACGTAAAGTATGGCAATCTTCTGAACGCGCAGCAGGCGTCGAACAACAAGTACGT
TCCTCGTGCCGTCCTCGTCGACCTCGAGCCCGGCACCATGGATGCCGTCCGCGCCGGCCCCTTCGGCCAGCTC
TTCCGCCCCGACAACTTTGTCTTCGGCCAGTCTGGTGCCGGTAACAACTGG

>OQ091958_CREA-DC TPR OL.438 TUB2
ACCTGCGCCTGCCCGCTTGTTTTTCTACGCGTCCCCTGACAGCCCCGCGTCTTGCCCCCCGGCTACGACAACG
GGGTCAACACCGCTGCGCACTCCTGCTAACGTCGTCTTTTCGCATCCATAGGTTCACCTCCAGACCGGCCAAT
GCGTAAGTCTCCTCACATCCGCTGGAATCGCTGCATCGCGCTGACTTTGCCCAGGGTAACCAAATCGGTGCTG
CCTTCTGGTTTGTTGCCAAAACACTGCCGCTCCCGCGCCCCCGCTGACGCGAATCGACACCGCAGGCAGACCA
TTTCTGGCGAGCACGGCCTGGACGGCTCTGGCGTGTAAGTCTGCGCCGTTTCTTGCGCGAATGGCAATGGCTG
ACCCGCAGCAGCTACAATGGCACCTCCGACCTCCAGCTCGAGCGCATGAACGTCTACTTCAACGAGGTACTCT
CTCACTAATCGCACAAACACGTAAAGTATGGCAATCTTCTGAACGCGCAGCAGGCGTCGAACAACAAGTACGT
TCCTCGTGCCGTCCTCGTCGACCTCGAGCCCGGCACCATGGATGCCGTCCGCGCCGGCCCCTTCGGCCAGCTC
TTCCGCCCCGACAACTTTGTCTTCGGCCAGTCTGGTGCCGGTAACAACTGG

>OQ091959_CREA-DC TPR OL.453 TUB2
ACCTGCGCCTGCCCGCTTGTTTTTCTACGCGTCCCCTGACAGCCCCGCGTCTTGCCCCCCGGCTACGACAACG
GGGTCAACACCGCTGCGCACTCCTGCTAACGTCGTCTTTTCGCATCCATAGGTTCACCTCCAGACCGGCCAAT
GCGTAAGTCTCCTCACATCCGCTGGAATCGCTGCATCGCGCTGACTTTGCCCAGGGTAACCAAATCGGTGCTG
CCTTCTGGTTTGTTGCCAAAACACTGCCGCTCCCGCGCCCCCGCTGACGCGAATCGACACCGCAGGCAGACCA
TTTCTGGCGAGCACGGCCTGGACGGCTCTGGCGTGTAAGTCTGCGCCGTTTCTTGCGCGAATGGCAATGGCTG
ACCCGCAGCAGCTACAATGGCACCTCCGACCTCCAGCTCGAGCGCATGAACGTCTACTTCAACGAGGTACTCT
CTCACTAATCGCACAAACACGTAAAGTATGGCAATCTTCTGAACGCGCAGCAGGCGTCGAACAACAAGTACGT
TCCTCGTGCCGTCCTCGTCGACCTCGAGCCCGGCACCATGGATGCCGTCCGCGCCGGCCCCTTCGGCCAGCTC
TTCCGCCCCGACAACTTTGTCTTCGGCCAGTCTGGTGCCGGTAACAACTGG

DNA-directed RNA polymerase II second largest subunit (RPB2)

>OQ091960_CREA-DC TPR OL.60 RPB2
TCCTGATGTGCAACGTCACCGTCGGTAGCGACGTCACTCCCATTCAGGATTTCATGACCCAGCGGAACATGGA
GCTTCTGGAAGAGTACGAGCCGAATGTCTCGCCCAATGCCACTAAGATTTTCATCAACGGTGTCTGGGTTGGT
GTTCATCGTGACCCAACCCAGCTCGTCTCCGTCGTTAAGAAGCTGCGCCGAGACGGCACCCTGTCTGCGGAAA
TGAGTCTTATTCGCGATGTCCGTGACAGGGAGTTCAAGATTTTCACCGATGCAGGTCGAGTGTGCAGGCCCCT
CTTCATCATTGACGACGATCCATTCAGCCCGAATAAGGGGAACTTGGTCTTGGCCCGAGAGCACATCGACAAA
CTGGAGGCAGATCAAGAAATCGACGTTTCTGGAATGAACGATGACGAAAGAGATGAGAAGAGGTATGGCTGGA
AGGGGTTACTGCAGAGCGGTGTGGTCGAGTACATGGATGCGGAAGAAGAGGAGGTTGCGATGATTACCATGAC
TCCCGACGATTTGAGGGCGCACCACCGAGCTCGTCAAGGCATCATCGATGAAGAAGACGAAGAGAGCAAGCGC
AACAGGGATCCTCACGAGCGTGTCGTCCCGGCCCCCAACCCAAGTGTCAAGCAGTACACGCATTGCGAGATCC
ATCCCAGTATGATTCTCGGAATTTGTGCCAGCATCATTCCGT

>OQ091961_CREA-DC TPR OL.431 RPB2
TCCTGATGTGCAACGTCACCGTCGGTAGCGACGTCACTCCCATTCAGGATTTCATGACCCAGCGGAACATGGA
GCTTCTGGAAGAGTACGAGCCGAATGTCTCGCCCAATGCCACTAAGATTTTCATCAACGGTGTCTGGGTTGGT
GTTCATCGTGACCCAACCCAGCTCGTCTCCGTCGTTAAGAAGCTGCGCCGAGACGGCACCCTGTCTGCGGAAA
TGAGTCTTATTCGCGATGTCCGTGACAGGGAGTTCAAGATTTTCACCGATGCAGGTCGAGTGTGCAGGCCCCT
CTTCATCATTGACGACGATCCATTCAGCCCGAATAAGGGGAACTTGGTCTTGGCCCGAGAGCACATCGACAAA
CTGGAGGCAGATCAAGAAATCGACGTTTCTGGAATGAACGATGACGAAAGAGATGAGAAGAGGTATGGCTGGA
AGGGGTTACTGCAGAGCGGTGTGGTCGAGTACATGGATGCGGAAGAAGAGGAGGTTGCGATGATTACCATGAC
TCCCGACGATTTGAGGGCGCACCACCGAGCTCGTCAAGGCATCATCGATGAAGAAGACGAAGAGAGCAAGCGC
AACAGGGATCCTCACGAGCGTGTCGTCCCGGCCCCCAACCCAAGTGTCAAGCAGTACACGCATTGCGAGATCC
ATCCCAGTATGATTCTCGGAATTTGTGCCAGCATCA

>OQ091962_CREA-DC TPR OL.438 RPB2
CAACGTCACCGTCGGTAGCGACGTCACTCCCATTCAGGATTTCATGACCCAGCGGAACATGGAGCTTCTGGAA
GAGTACGAGCCGAATGTCTCGCCCAATGCCACTAAGATTTTCATCAACGGTGTCTGGGTTGGTGTTCATCGTG
ACCCAACCCAGCTCGTCTCCGTCGTTAAGAAGCTGCGCCGAGACGGCACCCTGTCTGCGGAAATGAGTCTTAT
TCGCGATGTCCGTGACAGGGAGTTCAAGATTTTCACCGATGCAGGTCGAGTGTGCAGGCCCCTCTTCATCATT
GACGACGATCCATTCAGCCCGAATAAGGGGAACTTGGTCTTGGCCCGAGAGCACATCGACAAACTGGAGGCAG
ATCAAGAAATCGACGTTTCTGGAATGAACGATGACGAAAGAGATGAGAAGAGGTATGGCTGGAAGGGATTACT
GCAGAGCGGTGTGGTCGAGTACATGGATGCGGAAGAAGAGGAGGTTGCGATGATTACCATGACTCCCGACGAT
TTGAGGGCGCACCACCGAGCTCGTCAAGGCATCATCGATGAAGAAGACGAAGAGAGCAAGCGCAACAGGGATC
CTCACGAGCGTGTCGTCCCGGCCCCCAACCCAAGTGTCAAGCAGTACACGCATTGCGAGATCCATCCCAGTAT
GATTCTCGGAATTTGTGCCAGCATCAT

>OQ091963_CREA-DC TPR OL.453 RPB2
CTGATGTGCAACGTCACCGTCGGTAGCGACGTCACTCCCATTCAGGATTTCATGACCCAGCGGAACATGGAGC
TTCTGGAAGAGTACGAGCCGAATGTCTCGCCCAATGCCACTAAGATTTTCATCAACGGTGTCTGGGTTGGTGT
TCATCGTGACCCAACCCAGCTCGTCTCCGTCGTTAAGAAGCTGCGCCGAGACGGCACCCTGTCTGCGGAAATG
AGTCTTATTCGCGATGTCCGTGACAGGGAGTTCAAGATTTTCACCGATGCAGGTCGAGTGTGCAGGCCCCTCT
TCATCATTGACGACGATCCATTCAGCCCGAATAAGGGGAACTTGGTCTTGGCCCGAGAGCACATCGACAAACT
GGAGGCAGATCAAGAAATCGACGTTTCTGGAATGAACGATGACGAAAGAGATGAGAAGAGGTATGGCTGGAAG
GGGTTACTGCAGAGCGGTGTGGTCGAGTACATGGATGCGGAAGAAGAGGAGGTTGCGATGATTACCATGACTC
CCGACGATTTGAGGGCGCACCACCGAGCTCGTCAAGGCATCATCGATGAAGAAGACGAAGAGAGCAAGCGCAA
CAGGGATCCTCACGAGCGTGTCGTCCCGGCCCCCAACCCAAGTGTCAAGCAGTACACGCATTGCGAGATCCAT
CCCAGTATGATTCTCGGAATTTGTGCCAGCATCATTCCGTTCCCCGATCAC
